# Supplementary material for: RNAi-Mediated Knockdown of Cottontail Rabbit Papillomavirus Oncogenes Using Low-Toxicity Lipopolyplexes as a Paradigm to Treat Papillomavirus-Associated Cancers
Source: Pharmaceutics. 2023 Sep 25;15(10):2379. doi: 10.3390/pharmaceutics15102379 (PMC10610439; doi:10.3390/pharmaceutics15102379)
Supplement: Supplementary file 1 [file pharmaceutics-15-02379-s001.zip › pharmaceutics-2545136-supplementary.pdf]

Supplementary Materials

Table S1. Oligo Primers

Primer ID	*Primer sequence*	*Primer ID*	*Primer sequence*
GAPDH_1f	5'-AAGGTCATCCACGACCACTT-3'	GAPDH_1r	5'-GTCTTCTGGGTGGCAGTGAT-3'
GAPDH_2f	5'-CTGCACCACCAACTGCTTAG-3'	GAPDH_2r	5'-GTCTTCTGGGTGGCAGTGAT-3'
rRPLPO_1f	5'-GAAGCTGCTGCCTCATATCC -3'	rRPLPO_1r	5'-GGCCAGTAGCATATCCCTGA-3'
rRPLPO_2f	5'-CGACGTGCAGCTGATAAAGA-3'	rRPLPO_2r	5'-GAGAAGGGCGAGATGTTCAG-3'
rRPL32_1f	5'-CGGAAACCCAGAGGTATTGA-3'	rRPL32_1r	5'-GCATGTGCTTGGTCTTCTTG-3'
rRPL32_2f	5'-TAAGCGTAACTGGCGGAAAC-3'	rRPL32_2r	5'-GCATGTGCTTGGTCTTCTTG-3'
CRPV_E6_1f	5'-GGACTTGCCGTTTGGTTGTA-3'	CRPV_E6_1r	5'-ATAGCCCCGTGCATTTGA-3'
CRPV_E6_2f	5'-GCTAGAGAAGCTGCAGCAAA-3'	CRPV_E6_2r	5'-ATAGCCCCGTGCATTTGA-3'
CRPV_E7_1f	5'-CATTGCGACGAAGCATTAGA-3'	CRPV_E7_1r	5'-CTTACATGGCACGGACACTG-3'
CRPV_E7_2f	5'-CAGTGTCCGTGCCATGTAAG-3'	CRPV_E7_2r	5'-ATGCGGATAGCAGTCGATTC-3'

(f = forward, r = reverse)

Table S2. List of primer pairs containing restriction sites for the generation of CRPV E6 & E7 expression plasmids.

DNA construct	*Direction*	*Primer pair*	*Restriction sites*	*Amplicon size (bp)*
E6_wt	forward	5'-GATCGA*AAGCTT*GCCACCATGGAGAACTGCCTGCC-3'	*HindIII*	852
	reverse	5'-AGCTAG*GAATTC*TCATCTAAATTCTGTGAAG-3'	*EcoRI*	
E7_wt	forward	5'-GATCGA*GGATCC*GCCACCATGATAGGCAGAACTCCTAAG-3'	*BamHI*	315
	reverse	5'-AGCTAG*GAATTC*TCAGTTACAACACTCCGGGC-3'	*EcoRI*	
E6_G/R	forward	5'-CAGTCA*CTCGAG*CTATGGAGAACTGCCTGCCACGC-3'	*XhoI*	848
	reverse	5'-AGCTAG*GAATTC*TCATCTAAATTCTGTGAAG-3'	*EcoRI*	
E7_G/R	forward	5'-GATCGA*CTCGAG*CTATGATAGGCAGAACTCCTAAG-3'	*XhoI*	311
	reverse	5'-AGCTAG*GAATTC*TCAGTTACAACACTCCGGGC-3'	*EcoRI*	

(wt= wilde type, G=GFP, R=RFP)

Table S3. siRNA Sequences* (Dharmacon Inc, USA)

siRNA ID	*siRNA Sequence*
CRPV_E6_siRNA1	5'-CGGAACAGGTGATACAGAT-3'
CRPV_E6_siRNA2	5'-CGTTGTACAGTTTGCGGAA-3'
CRPV_E6_siRNA3	5'-CGTTGTACAGTTTGCGGAA-3'
CRPV_E7_siRNA1	5'-TGTAAGCGCTGTAGGCAAATT-3'
CRPV_E7_siRNA2	5'-GCGACGAAGCATTAGAGAATT-3'
CRPV_E7_siRNA3	5'-GGAGGATCATCAAGATAGATT-3'

*shown is the respective DNA sequence

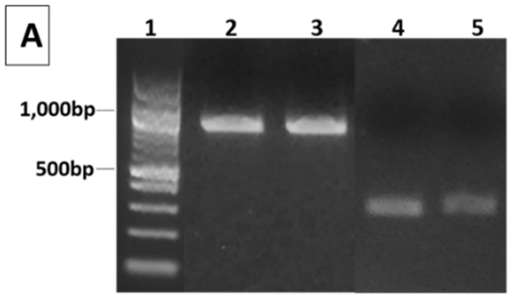

Lane No	DNA amplicons with RE sites	Amplicon size (bp)
2	E6_w (HindIII / EcoRI)	852
3	E6 _ G/R (XhoI / EcoRI)	848
4	E7 _ G/R (XhoI / EcoRI)	311
5	E7_w (BamHI / EcoRI)	315
1	100 bp DNA ladder	

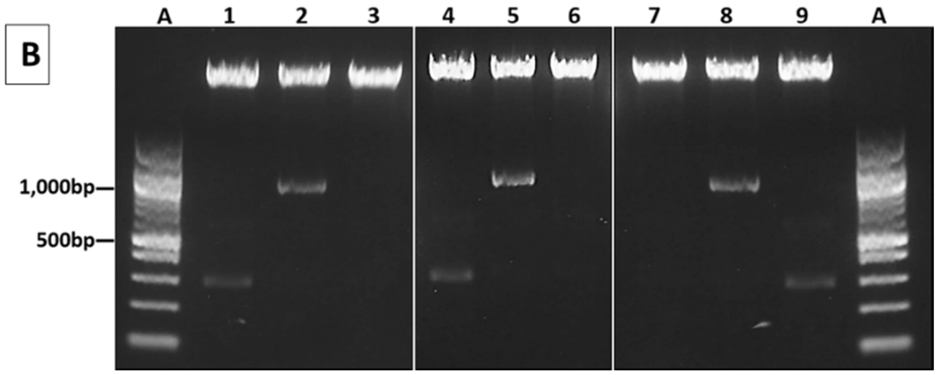

Figure S1. Cloning of CRPV E6/E7 expression constructs. (A) Gel showing PCR amplification of full gene length products (E6 and E7 amplicons) with different restriction sites for cloning into pcDNA 3.0, pEGFP-C1, and pDsRed-monomer-N1 vectors. Lane number, amplicons with restriction sites and their sizes are mentioned in table next to gel electrophoresis image. (B). Recombinant plasmids verification by restriction endonucleases digestion. Lane A: 100bp DNA ladder; Lane 1: HindIII and EcoRI digested E6_WT clone; Lane 2: BamHI and EcoRI digested E7_WT plasmid; Lane 4: XhoI and EcoRI digested GFP_E7 plasmid; Lane 5: XhoI and EcoRI digested GFP_E6 plasmid; Lane 8: XhoI and EcoRI digested RFP_E6 plasmid; Lane 9: XhoI and EcoRI digested RFP_E7 plasmid; Lane 3, 6, 7: RE digestion of non-recombinant plasmids without DNA inserts yield bands of pcDNA 3.0, EGFP, DsRed-Monomer-C respectively.

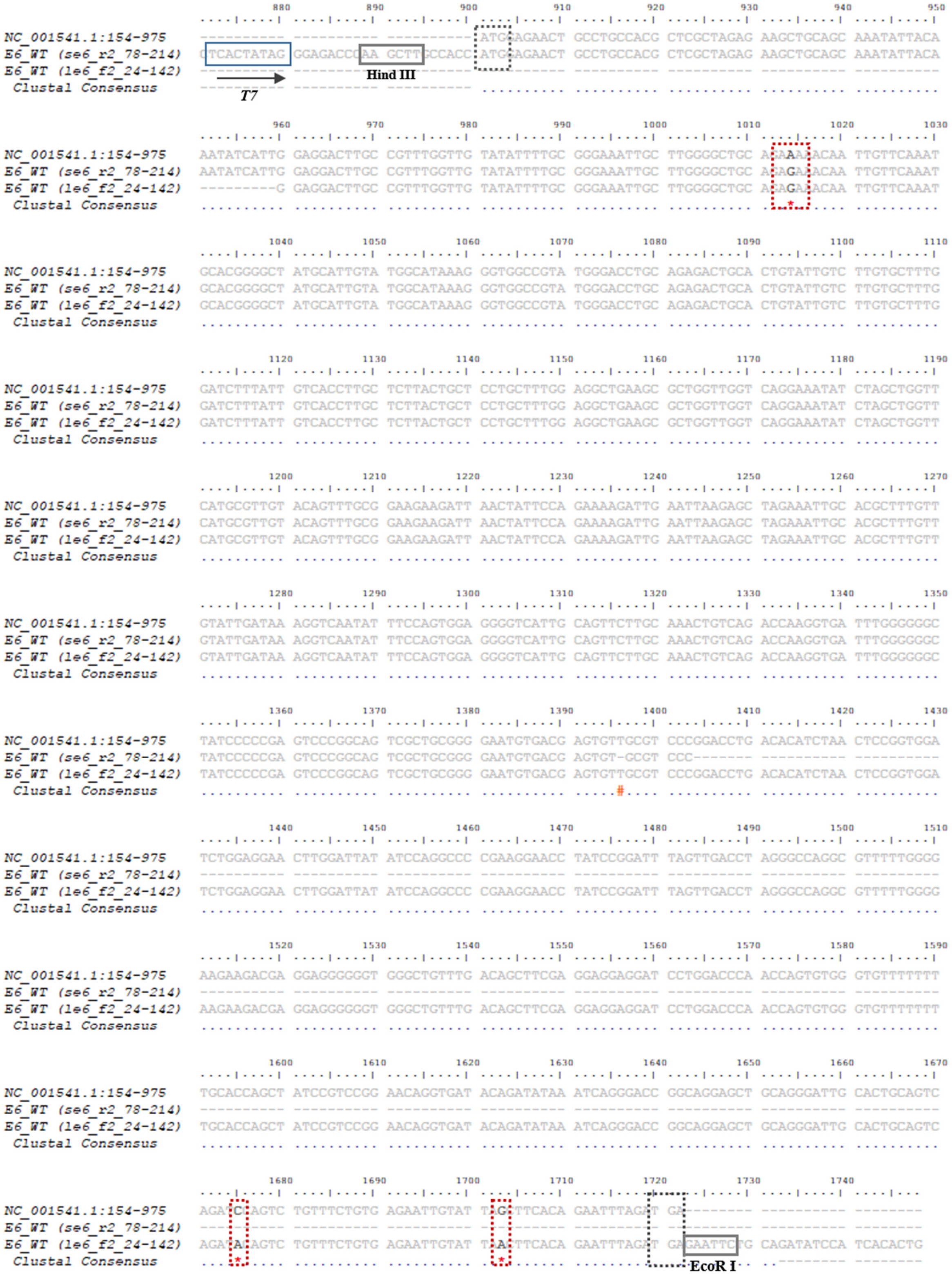

Figure S2. Sequence alignment of the E6_WT clone. Analysis of sequence alignment to predicted CRPV reference sequence (*NC_001541.1:154-975*) identified nucleotide mismatches at position 1014, 1675 & 1703 (marked by box with red dotted lines). Initiation and termination codons are mentioned in grey dotted box. Sequence also confirmed sequences of restriction sites. T7 promoter sequences provides confirmation of pcDNA 3.0 vector. (#=missing nucleotide only in one direction, sequence correct). (This construct was not used in this study)

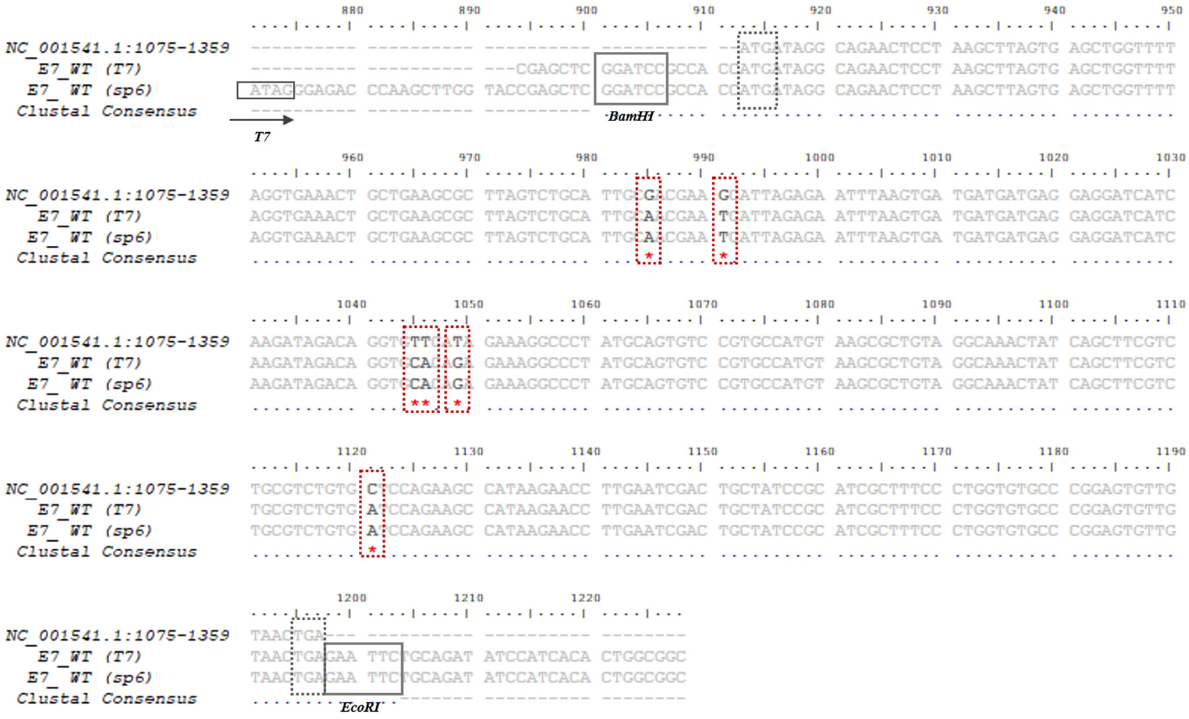

Figure S3. Sequence alignment of the E7_wt clone to the predicted CRPV reference sequence (*NC_001541.1:1075-1359*) identified nucleotide mismatches at position 985, 991, 1045, 1046, 1049 and 1121 (marked by box with red dotted lines). Start and stop codons are highlighted in grey dotted boxes. Also depicted are the introduced restriction sites BamHI and EcoRI. (This construct was not used in this study)

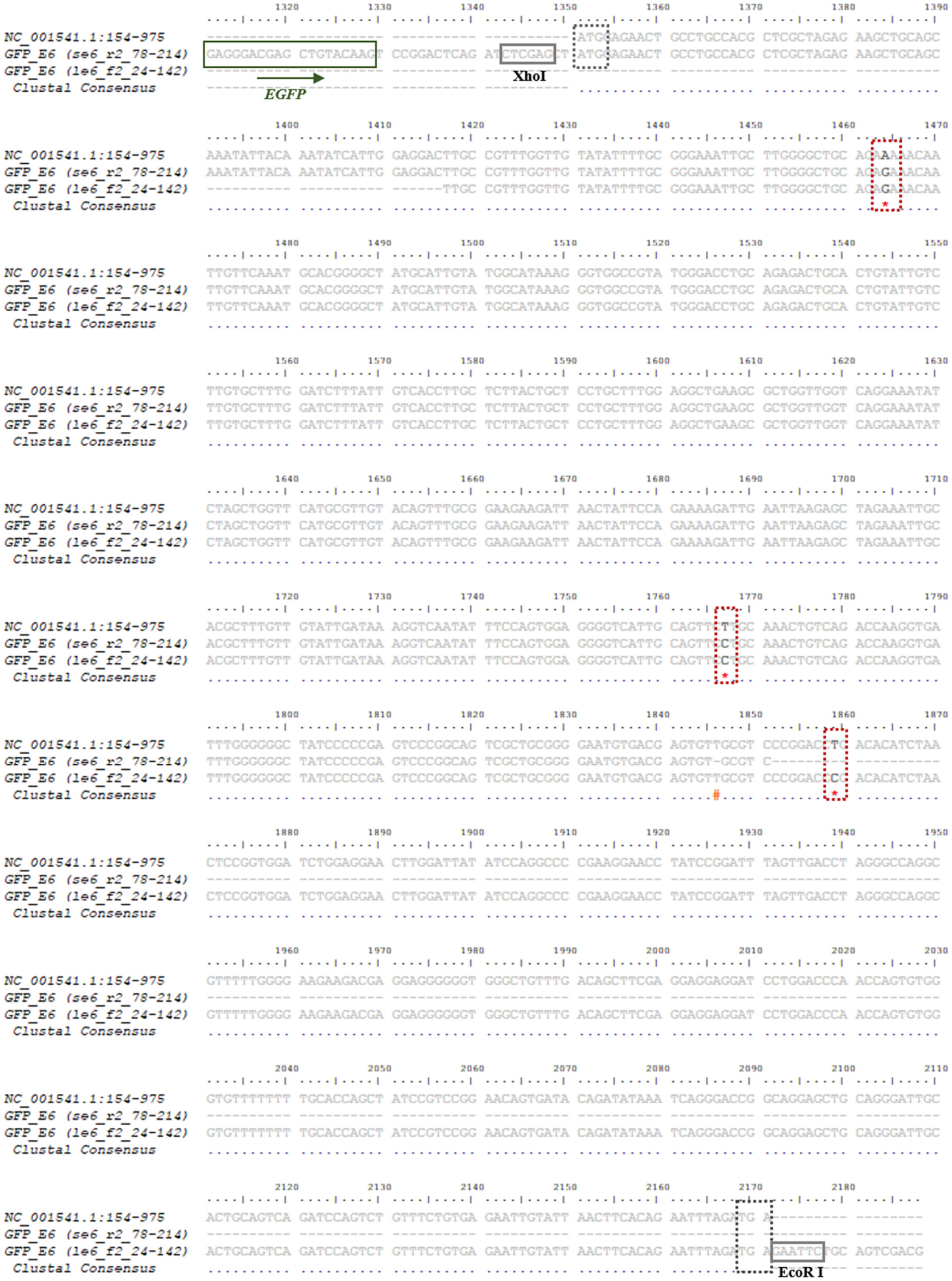

Figure S4. Sequence alignment of the GFP_E6 clone. Analysis of sequence alignment to predicted CRPV reference sequence (*NC_001541.1:154-975*) identified nucleotide mismatches at position 1464, 1767 & 1859 (marked by box with red dotted lines). Start and stop codons are mentioned in grey dotted box. Sequence also confirmed sequences of restriction sites. EGFP promoter sequences provides confirmation of pEGFP-C1 vector. One base deletion observed and marked with. (#: missing nucleotide only in one direction = sequence correct)

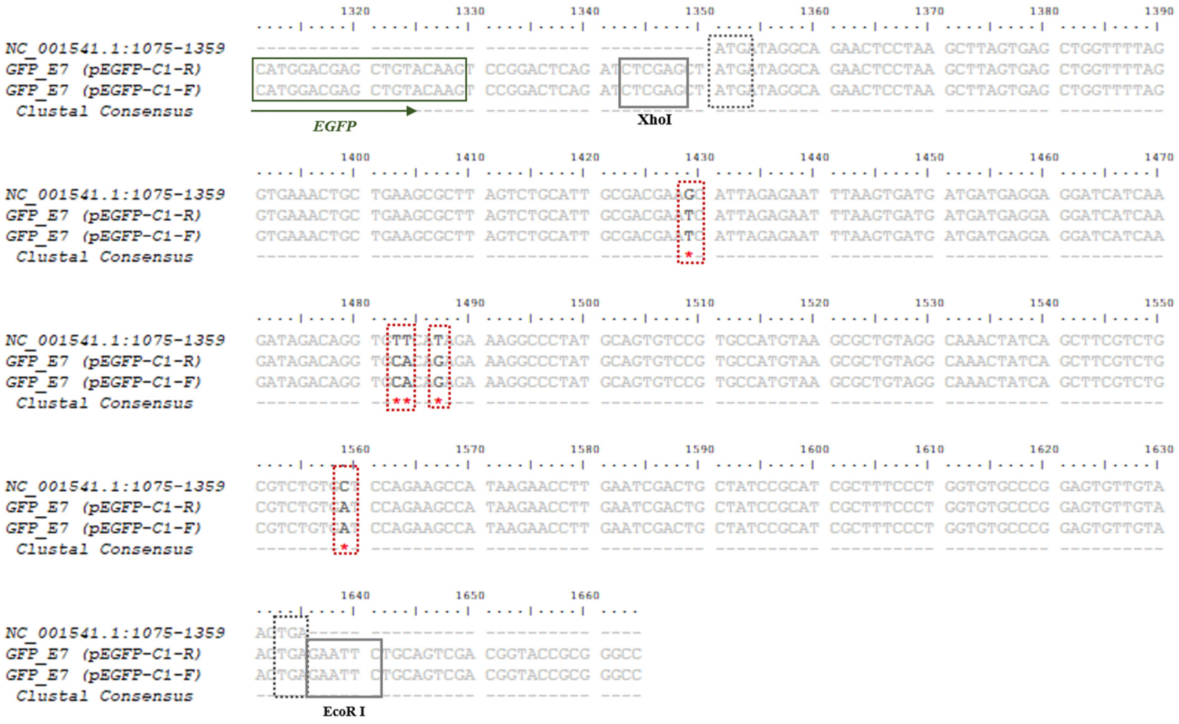

Figure S5. Sequence alignment of the GFP_E7 clone. Identification of nucleotide mismatches at position 1429, 1483, 1484, 1487, 1559 (marked by box with red dotted lines). Start and stop codons are highlighted in grey dotted boxes. Also depicted are the introduced restriction sites XhoI and EcoRI.

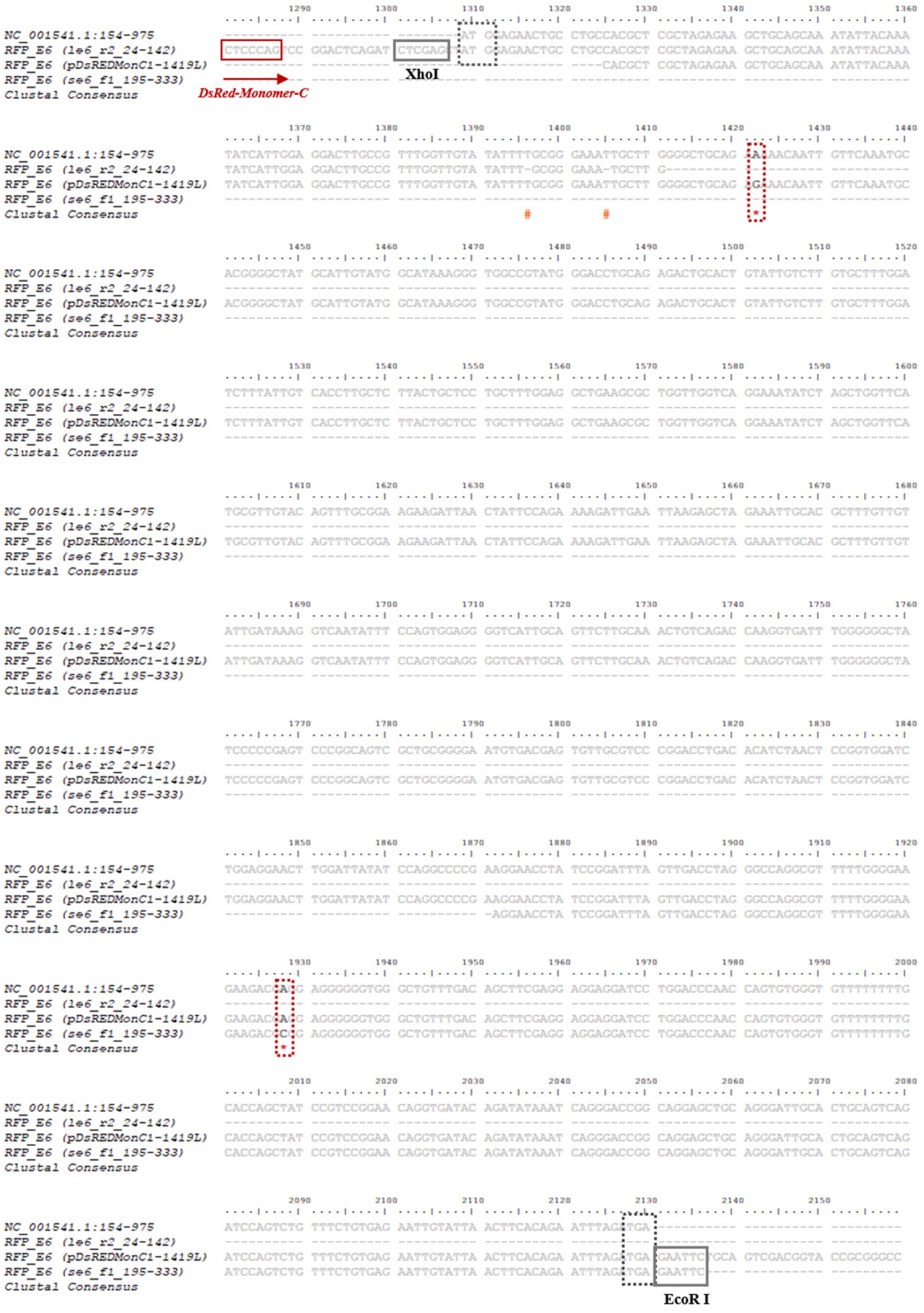

Figure S6. Sequence alignment of the RFP_E6 clone. Analysis of sequence alignment to predicted CRPV reference sequence (*NC_001541.1:154-975*) identified nucleotide mismatches at position 1422 & 1928 (marked by box with red dotted lines). Start and stop codons are mentioned in grey dotted box. Sequence also confirmed sequences of restriction sites. The promoter sequences provides confirmation of pDsRed-Monomer-C vector. (#: missing nucleotide only in one direction = sequence correct)

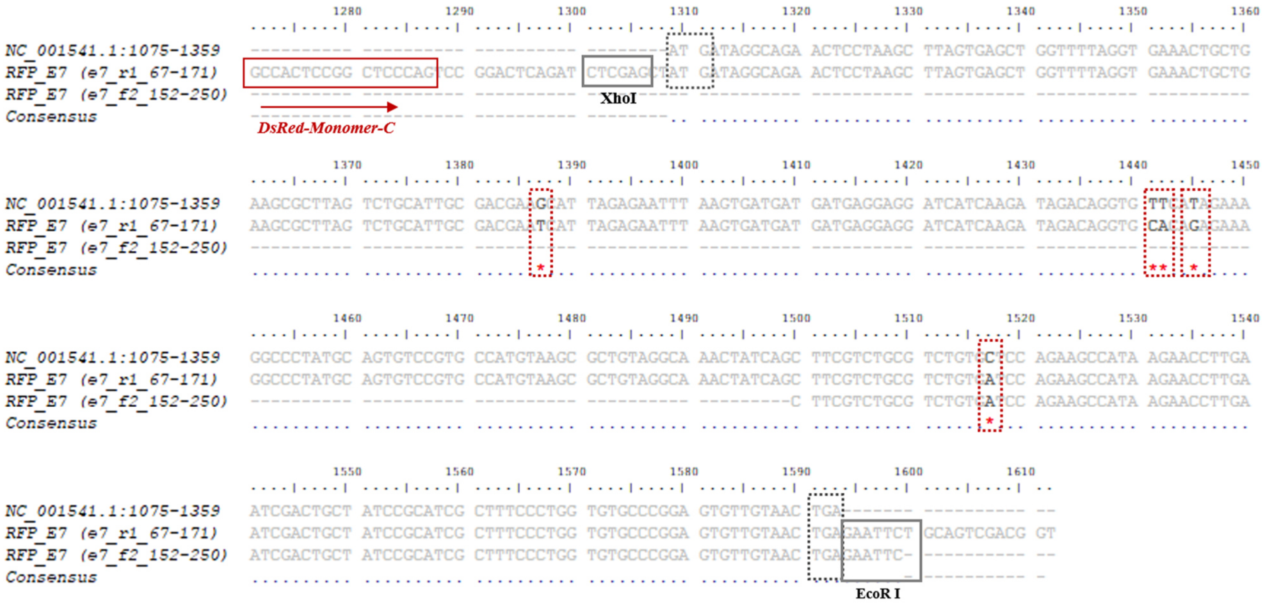

Figure S7. Sequence alignment of the RFP_E7 clone. Identification of nucleotide mismatches at position 1387, 1441, 1442, 1445 & 1517 (marked by box with red dotted lines). Start and stop codons are highlighted in grey dotted boxes. Also depicted are the introduced restriction sites XhoI and EcoRI.
